# Owens–Wendt Method for Comparing the UV Stability of Spontaneous Liquid-Repellency with Wet Chemical Treatment of Laser-Textured Stainless Steel

**DOI:** 10.3390/biomimetics8080584

**Published:** 2023-12-02

**Authors:** Oleksiy Myronyuk, Denys Baklan, Aleksej M. Rodin

**Affiliations:** 1 Department of Chemical Technology of Composite Materials, Chemical Technology Faculty, Igor Sikorsky Kyiv Polytechnic Institute, Beresteiskyi Ave. 37, 03056 Kyiv, Ukraine; o.myronyuk@kpi.ua (O.M.); d.baklan@kpi.ua (D.B.); 2 Solid State Laser Laboratory, Department of Laser Technologies, Center for Physical Sciences and Technology, Savanoriu Ave. 231, 02300 Vilnius, Lithuania

**Keywords:** UV stability, stainless steel, liquid-repellency, laser microprocessing, LIPSS, wet chemical treatment, Owens–Wendt approach

## Abstract

The liquid-repellent properties of AISI 304 stainless steel surfaces textured with a femtosecond laser were studied, both after spontaneous hydrophobization and when treated with stearic acid and octyltrimethoxysilane. Surface topography has been shown to play a critical role in determining these properties. Although textures containing only LIPSS exhibited poor liquid-repellency, the performance was significantly improved after engraving the microtexture. The most effective topography consisted of 45 µm-wide grooves with a pitch of 60 µm and protrusions covered with a rough microcrystalline structure. Liquid-repellency, chemical treatment efficiency, and UV resistance were compared using derived Owens–Wendt parameters. The surface of femtosecond-laser-textured steel after spontaneous hydrophobization was found to be significantly less stable under UV irradiation than surfaces treated with stearic acid or octyltrimethoxysilane modifiers.

## 1. Introduction

One of the striking examples of the transfer of the principles of biomimetics to materials science are surfaces with controlled wettability, in particular, enhanced water-repellency. The carriers of this natural property are lotus leaves, duck feathers, butterfly wings, and rose petals [1]. Liquid-repellency is vital for small organisms, the effect of surface tension on which can be a serious immobilizing factor [2]. The increased water-repellency of these natural surfaces is due to the developed relief, as well as the low intrinsic polarity of the surface, which causes a reduced surface energy [3]. The texture on the surface of natural formations is caused by various microscale protrusions, such as the tufts on the wings of a mosquito or the outgrowths on lotus leaves or Dahlia flower leaves, which usually contain fine-scale roughness formed by crystals of waxy substances [4]. In addition to the formation of a bi-hierarchical topography structure, which is considered the most effective in terms of the manifestation of water-repellent properties [5], this also provides a reduced intrinsic surface energy, since these waxes are relatively nonpolar hydrocarbon substances.

The use of similar principles to reduce the wetting of surfaces by various liquids opens up the possibility of obtaining materials with an increased resistance to icing [6], self-cleaning from dust and other contaminants under the water flow during rain [7,8], anticorrosion ability by reducing the fraction of interfacial surface contact [9,10], and anti-fogging ability due to condensation and the merging of liquid droplets [11,12,13]. Existing methods to impart micro- and nanotextures to surfaces, such as electrochemical deposition [14], chemical vapor deposition [15,16], wet chemical treatment [17,18], or sol-gel synthesis [19,20] are hampered by the high cost and the difficulty of scaling [21,22,23,24]. The use of laser texturing is one of the promising solutions to these challenges [25], at least for metal surfaces.

Femtosecond laser processing provides reproducible micro- and nanotextures, the latter known as laser-induced periodic surface structures (LIPSS). This method can be successfully applied to the steel surface [26], and the resulting texture parameters affect the water-repellency properties [27]. Bi-hierarchical surfaces with overlapping microtexture and LIPSS have proven to be more effective [28]. In this case, additional treatment is usually carried out to reduce the surface energy [29], resulting in water contact angle values exceeding 150°, which corresponds to a superhydrophobic state [30]. For such post-treatment, chemical vapor deposition [31], chemical etching [32], the sol-gel method [33], and chemical functionalization [34] can be used.

It is noteworthy that after the laser texturing of metals, a non-obvious phenomenon of spontaneous surface hydrophobization was observed. The essence of this phenomenon lies in the wetting transition, which can be tracked by changes in water contact angles from values less than 30° to more than 150° simply by prolonged exposure to air. The effect was observed for a wide variety of metal surfaces: steel [35,36], aluminum alloy [37], titanium [38], copper [39], etc. This phenomenon is explained by the action of carbon dioxide [40,41], water from the surrounding atmosphere [42], or, most likely, airborne hydrocarbons [43]. As was shown in [44], this effect may be reproduced by keeping samples in an atmosphere with polar volatile organic acids. Spontaneous hydrophobization may seem to be a practically useful phenomenon, since it allows one to obtain surfaces with reduced wettability without the need for subsequent treatment. However, the wait to achieve the desired level of properties may take longer than a month [45]. Since the nature of spontaneous hydrophobization is not yet fully understood, and may depend on the atmosphere exposed to the sample after treatment, the influence of surface topography and its development remains unpredictable. The disadvantages of this phenomenon also include the further instability of the resulting surface layer to environmental factors [22]. However, most studies on the durability of superhydrophobic coatings have focused on corrosion resistance [46,47,48] and mechanical [49] and chemical resistance [50], emphasizing the importance of the chemical treatment agent. Meanwhile, UV radiation is also a significant environmental factor, which is known to cause the degradation of organic hydrocarbons [51]. Over time, under the influence of UV irradiation, such layers lose their ability to repel liquids [52] due to the formation of polar surface groups during photodegradation. This factor was considered in [53], and myristic acid was shown to be effective as a treatment agent. Obviously, long-term UV resistance is key when deploying textured metals after hydrophobization outdoors. Meanwhile, natural formations remain hydrophobic even after prolonged exposure to sunlight, spurring improvements in both laser processing technologies and chemical post-treatment.

Thus, the purpose of this work was to clearly compare the UV resistance of an organic layer formed as a result of spontaneous hydrophobization and layers obtained by wet chemical treatment of the same surfaces. Surface energy modifiers of femtosecond-laser-textured AISI 304 stainless steel [54,55,56] were selected from the groups of carbon- and silane-based treatment agents. The modified Owens–Wendt approach we described in [57] was used as a tool to compare the polarity (and resulting liquid-repellent properties) of textures after spontaneous hydrophobization and to track the polarity changes of textured surfaces after reported chemical treatments. The study examined textures obtained under different laser-processing regimes: from LIPSS alone or microgrooves to combinations of both. This made it possible to identify the influence of their geometric features on the wetting characteristics after spontaneous hydrophobization.

## 2. Materials and Methods

### 2.1. Femtosecond Laser Texturing

The smooth surface of 20 mm × 20 mm AISI 304 stainless steel plates with a thickness of 2 mm was degreased and cleaned in isopropyl alcohol before laser processing. The experimental setup has been reported and discussed in earlier publications [52,58]. The sample surface was processed with a Carbide laser (Light Conversion, Vilnius, Lithuania), providing a pulse width of ~360 fs at a wavelength of 1030 nm. With a fixed repetition rate of 60 kHz used in all experiments, the average power was limited to ~3 W. The sample movement speed was maintained at 6 cm/s.

Laser processing parameters for LIPSS and microgrooves were optimized to provide the best performance and minimal sample contamination. LIPSS patterns were formed by focusing a laser beam with an aspheric lens of 100 mm focal length into a spot with a diameter of ~47 µm (FWHM) and a peak fluence of ~1.83 J/cm^2^. The laser spots overlapped by ~60% during scanning and the pitch of adjacent passes was 30 µm. To process microgrooves, the laser beam after the Galilean telescope was expanded 4 times and focused into a spot with a diameter of ~3 µm (FWHM). Microgrooves were machined in 15 consecutive passes to a depth of ~20 µm. The grooves on the samples “6030L” and “10030L” were processed at a peak fluence of ~214 J/cm^2^, and on the samples “6045L” and “6045” at a peak fluence of ~357 J/cm^2^, respectively.

After the laser surface texturing, the samples were washed for 10 min in an ultrasonic bath, first in distilled water and then in ethyl alcohol. After this, the samples were heated for 1.5 h at 160 °C. The textured samples were then stored for 3 months at 22 °C and relative humidity ~50%. A stainless steel sample without applied texture was used as a reference.

### 2.2. Chemical Surface Treatment

**Figure 1:**
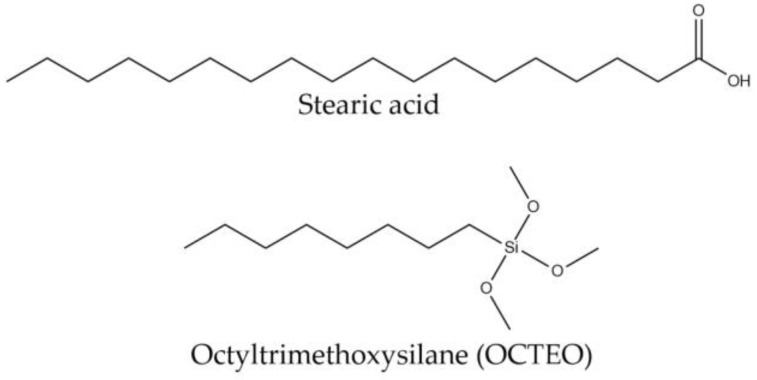
The structural formulas of modifiers used for surface treatment.

A common post-treatment option is chemical modification of the surface, for example with fluorosilanes [59,60]. However, octyltrimethoxysilane (OCTEO) (CAS 3069-40-7) was used in this study because it is more environmentally friendly compared to fluorinated analogs [61,62,63]. The second modifier used was stearic acid (CAS 57-11-4), which has an even lower environmental impact due to its natural origin and is also a carbon-based organic material closer in composition to the substance absorbed during spontaneous hydrophobization than siloxane. The structural formulas of the modifiers are presented in Figure 1.

To clean the substrates before chemical treatment, the samples were first heat-treated for 1 h at 400 °C, then washed in isopropyl alcohol and dried for 10 min at 80 °C. For stearic acid treatment, samples were immersed in a 0.5 wt.% solution in isopropyl alcohol for half an hour at room temperature, followed by oven drying at 50 °C for half an hour. For OCTEO treatment, samples were immersed in a 1 wt.% solution of the modifier in isopropyl alcohol for half an hour at room temperature and dried at 130 °C for half an hour, similar to the procedure described in [64].

### 2.3. Surface Characterization

The surface topography of textured samples was studied using a MIRA3 LMU scanning electron microscope (Tescan, s.r.o., Brno, Czech Republic). Contact angles were determined using an optical microscope, a digital camera, and the method described in [52] using a micropipette and droplets applied at five different points on the sample surface. After measuring the contact angle, the samples were dried for 60 s at 60 °C. To increase the measurement resolution, mixtures of water and ethanol were used as probe liquids. The surface tension of water–ethanol probe liquids was calculated using the dependences from [65]. The elemental composition of the textured surface was determined using an energy dispersive X-ray spectrometer (EDS) (INCA X-ACT from Oxford Instruments, Abingdon, UK).

### 2.4. UV Resistance Tests

During spontaneous hydrophobization, a nonpolar organic layer is adsorbed from the gas phase on the metal surface, providing it with water-repellent properties. Accordingly, the standard practices ASTM G154-23 (https://www.astm.org/g0154-23.html, accessed on 25 October 2023) and ASTM D4329-21 (Cycle A) (https://www.astm.org/d4329-21.html, accessed on 25 October 2023) were used for the accelerated aging of organic surfaces. A fluorescent lamp with an emission intensity of 0.7 W/m^2^ at a wavelength of 340 nm (UVA 340) was used as a source of UV light. To evaluate only the photoresistance of surfaces, the procedure was modified to eliminate the influence of humidity and there was no periodic irradiation. To prevent heating, the samples were placed on a steel heat sink at a distance of 170 mm from the UV lamp. After irradiation, the samples were cooled to room temperature for 5 min before measuring contact angles.

### 2.5. Determination of Surface Energy by the Owens–Wendt Method

The Owens–Wendt approach is based on the free surface energy Equation (1) [66]:

(1) $$\sigma_{SL}=\sigma_{S}+\sigma_{L}-2\left(\sqrt{\sigma_{S}^{D}\sigma_{L}^{D}}+\sqrt{\sigma_{S}^{P}\sigma_{L}^{P}}\right)$$

where $\sigma_{SL}$ is the surface energy at the solid-liquid interface; $\sigma_{S}$ is the surface energy at the solid–air interface; $\sigma_{L}$ is the surface tension of the liquid, and the indices *D* and *P* correspond to the disperse and polar components of the surface energy.

Combining this equation with Young’s equation and converting it to a linear form gives us the most practical Owens–Wendt Equation (2):

(2) $$\frac{\sigma_{L}(1+cos\theta )}{2\sqrt{\sigma_{L}^{D}}}=\frac{\sqrt{\sigma_{S}^{P}}\sqrt{\sigma_{L}^{P}}}{\sqrt{\sigma_{L}^{D}}}+\sqrt{\sigma_{S}^{D}}$$

where *θ* is the contact angle of the probe liquid (for certain $\sigma_{L}$, $\sigma_{L}^{D}$, and $\sigma_{L}^{P}$) with the tested surface.

**Figure 2:**
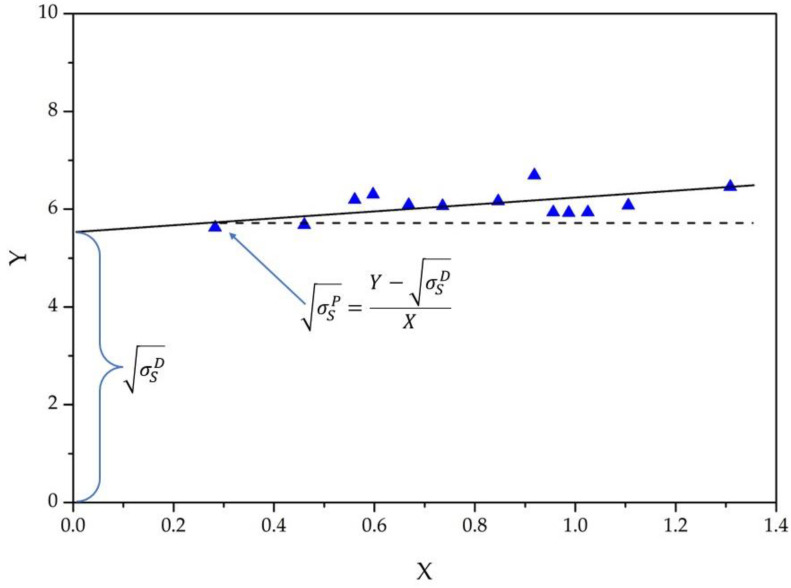
An example of an Owens–Wendt plot where the X axis is $\frac{\sqrt{\sigma_{L}^{P}}}{\sqrt{\sigma_{L}^{D}}}$ and the Y axis is $\frac{\sigma_{L}(1+cos\theta )}{2\sqrt{\sigma_{L}^{D}}}$.

The linear form provides the ability to solve equations graphically by plotting the Owens–Wendt graph, which is a straight line for flat surfaces, as shown in Figure 2 for a silane-treated steel surface. The X coordinates of all experimental points are defined solely by the polarity of the probe liquid, and the Y coordinates are defined by its surface tension and the contact angle value (obtained experimentally). The disperse component of the solid surface energy is defined as the intersection of the fitting line with the Y axis, and the polar component is defined by the slope of the fitting line and can be calculated as shown in Figure 2.

However, the classical Owens–Wendt approach is not applicable here, since it does not take into account the contribution of surface roughness, i.e., the occurrence of Cassie and Wenzel wetting states. We have shown [57] that such states cause distortions in the linear form of the Owens–Wendt diagram and can serve as a tool for characterizing the Cassie state, the stability of surface wetting, and the transition of wetting to the Wenzel state.

In addition, the parameters $\sigma_{TS}^{D}$/$\sigma_{S}^{D}$ and $\sigma_{LIN}^{P}$, related to the texture and chemical composition of the surface, were proposed to numerically describe the wettability of textured surfaces. The parameter $\sigma_{TS}^{D}$/$\sigma_{S}^{D}$ describes the ratio of the disperse component of a textured surface ($\sigma_{TS}^{D}$), obtained experimentally, to the disperse component of the surface energy of a blank surface of the same material without texture ($\sigma_{S}^{D}$). The parameter $\sigma_{LIN}^{P}$ represents the minimum value of the polar component of the surface energy at which the transition from the Cassie to the Wenzel state occurs. The parameter $\sigma_{LIN}^{P}$ is sensitive to the polarity of the surface layer, which reflects the liquid-repellency. The position of the transition point of the Owens–Wendt plot from a curve to a linear form is used to determine $\sigma_{LIN}^{P}$.

## 3. Results and Discussion

### 3.1. Topography of Textured Surfaces

**Figure 3:**
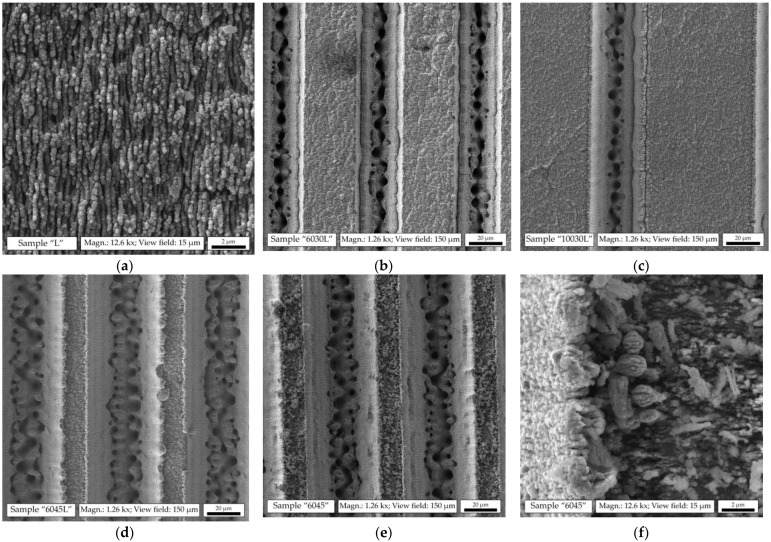
Sample surface topography under SEM: (**a**) LIPSS pattern on the sample “L”; (**b**) LIPSS on the sample “6030L” and 30 µm-wide grooves with a pitch of 60 µm; (**c**) LIPSS on the sample “10030L” and 30 µm-wide microgrooves with a pitch of 100 µm; (**d**) LIPSS on the sample “6045L” and 45 µm-wide microgrooves with a pitch of 60 µm; (**e**) 45 µm-wide microgrooves on the sample “6045” with a pitch of 60 µm; (**f**) crystal-like texture on the sample “6045” at high magnification.

The parameters of various micro- and nanotextures obtained on the surface of stainless steel after processing with femtosecond laser pulses are given in Table 1. The sample “L” contained only a nanoscale LIPSS pattern on the surface with a period of about 200 nm (Figure 3a). On the samples “6030L”, “10030L”, and “6045L”, a LIPSS pattern identical to the sample “L” was first formed, and only then were the microgrooves machined (Figure 3b–d). The samples “6045L” and “6045” (Figure 3d,e) exhibited an identical microtexture with a groove pitch of 60 µm and a width of 45 µm, but the sample “6045” did not contain the LIPSS pattern on top, although on the bevels of the grooves of the samples “6045” and “6045L” one can also notice a texture, quite similar to LIPSS, but less ordered. Such a pattern can be formed on the lateral inclined surfaces by the defocused peripheral part of the overlapping spots of the Gaussian laser beam when deepening the microgrooves during the final passes. In this case, the laser-machining regime was close to those necessary for the occurrence of LIPSS [60].

A protruding edge was observed along the walls of the grooves, especially pronounced in the samples “6045L” and “6045”. At the top of the sample “6045”, a rough texture with an element size of up to ~2 μm was observed (Figure 3f), which treatment in an ultrasonic bath did not allow to be washed off. This chaotic structure is formed by ejected metal solidified on a flat surface. However, the LIPSS pattern on the surface of the sample “6045L” prevented the adhesion of such a layer.

**Figure 4:**
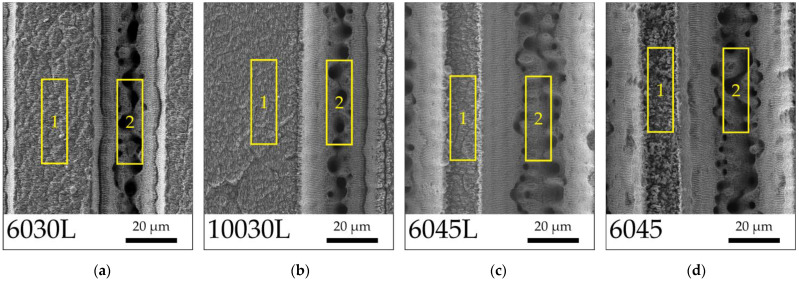
Texture scan areas for EDS analysis: (**a**) sample “6030L”; (**b**) sample “10030L”; (**c**) sample “6045L”, and (**d**) sample “6045”.

AISI 304 steel usually contains up to 0.08 wt.% carbon [67,68]; however, according to EDS-analysis (Figure 4, Table 2), an increased carbon content was observed on the textured surfaces, which was associated with the absorption of carbon-containing substances from the atmosphere. The areas at the top of the textures “1” and at the bottom of the microgrooves “2” were selected for scanning, respectively. While the carbon content at the top and bottom of the textures was similar for most samples with the exception of the sample “6045”, the oxygen content was significantly higher at the top, especially for the sample “6045”. This change is possibly associated with the oxidation of the upper part of the textured surface, while the chaotic crystal-like structure on the protrusions of the sample “6045” was subject to significant contamination with spontaneous hydrophobization substances. The oxidation of the protrusions areas “1” of the samples was higher than at the bottom of the grooves, possibly due to the absorption gradient.

### 3.2. Wetting the Sample Surface

**Figure 5:**
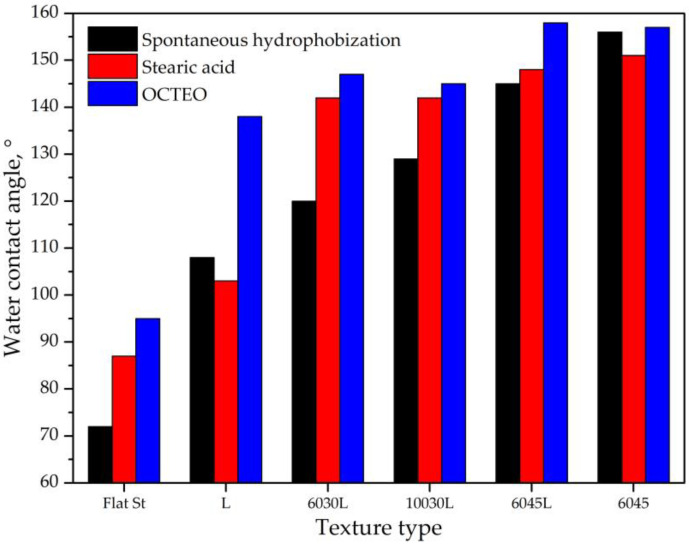
Wetting properties of textured surfaces before and after treatment compared to a reference plate (designated as “Flat St”).

The results of measuring the water contact angles of the obtained textures in comparison with the reference plate (designated as flat steel—“Flat St”) are shown in Figure 5. The blank reference sample had a contact angle of 72° before and 87° and 95° after the treatment with stearic acid and OCTEO, respectively.

Textured samples showed much higher water-repellency. In particular, for the samples “6045L” and “6045”, a superhydrophobic state was achieved with a contact angle of over 150°. Moreover, the laser texturing made it possible to obtain this state with all types of modifiers used, as well as for the spontaneously adsorbed layer. Notably, the sample “6045” did not contain a LIPSS nanopattern on top. In this case, it can be assumed that the increased water-repellency was due to the formation of an “acicular” crystalline structure on the microtexture protrusions (Figure 3f). The weakest water-repellency was demonstrated by the sample “L”, which contained only LIPSS, while the samples “6030L” and “10030L” were in the middle.

OCTEO was found to be the most effective modifier in reducing the surface energy for all samples. For the samples “L”, “6045L”, and “6045”, the effectiveness of stearic acid was close to that of the self-hydrophobizing layer. This turned out to be far from what was expected according to the model [44], which describes a higher nonpolarity of the long-chain absorbed acid layer. The sample “L” after self-hydrophobization had an even higher contact angle than with stearic acid, which may be due to the larger amount of the organic layer. For the samples “6030L”, “10030L”, and “6045L”, chemical modifiers provided a higher contact angle, while for the sample “6045L”, a superhydrophobic wetting state was achieved using OCTEO. In general, the sample “6045” demonstrated the best characteristics. It is noteworthy that the use of stearic acid for the samples “6045” and “L” even led to a slight decrease in the contact angle.

Thus, texture plays a decisive role in determining the maximum values of surface contact angle. In particular, the accompanying artifacts on the microtexture protrusions (on the sample “6045”) provided a greater contribution to water-repellency than the intentional LIPSS processing (for the samples “6030L”, “10030L”, and “6045L”). This is consistent with the results of [60] that the dimensionality of effective bi-hierarchical structures can differ from conventional micro- and nanotextures.

The chemical modifiers for the samples “6030L”, “10030L”, “6045L”, and “6045” had a similar efficiency, in most cases superior to spontaneous hydrophobization. This can be explained by the increased polarity of the self-hydrophobization layer and its small thickness [69,70].

### 3.3. Owens–Wendt Characterization

**Figure 6:**
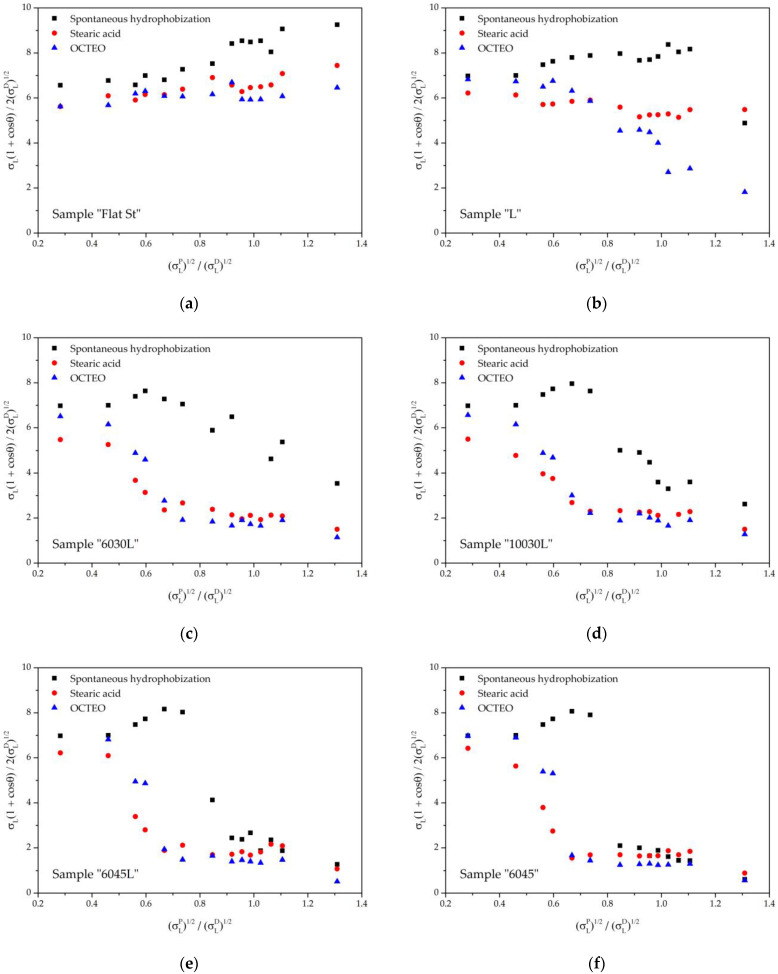
Owens–Wendt plots for reference plate and textured surfaces: (**a**) reference plate; (**b**) sample “L”; (**c**) sample “6030L”; (**d**) sample “10030L”; (**e**) sample “6045L”; and (**f**) sample “6045”.

To obtain the values of the surface energy component for a blank reference plate, an Owens–Wendt graph was plotted (Figure 6a). As follows from Table 3, the free surface energy of the sample after self-hydrophobization was the highest, and treatment with stearic acid and OCTEO made it possible to significantly reduce this value. The data obtained for stearic acid are consistent with the data in [71,72]. It is noteworthy that the values obtained with stearic acid and OCTEO treatments were quite similar. The Owens–Wendt plots for textured surfaces deviated from the linear form (Figure 6b–f). This deviation was more pronounced the more effective the modification was in terms of reducing the material’s intrinsic free surface energy.

The derivative parameter $\sigma_{TS}^{D}$/$\sigma_{S}^{D}$, indicating the minimum proportion of dispersion interaction of the texture surface compared to a flat surface (Table 4), is essentially an expression of the quality of water-repellency. Interestingly, the sample “L” with only the LIPSS nanopattern had the highest value, the samples with a combination of micro- and nanotextures “6030L” and “10030L” were in the middle, and the samples “6045L” and “6045” showed the lowest value.

In most cases, OCTEO-treated surfaces exhibited the greatest water-repellency, likely due to the achievement of the Cassie state, which implies only partial contact of the probe liquid. Spontaneously hydrophobized surfaces, on the contrary, showed a significant fraction of surface contact, which can be explained by the fact that the surface material was not sufficiently nonpolar to achieve the Cassie state and thus only the Wenzel state was realized. This was most obvious for the sample “L”.

The parameter $\sigma_{LIN}^{P}$ characterizes the value of the polar component of the probe liquid, below which the Owens–Wendt plot is linearized. Consequently, the higher this value, the lower the resistance of the surface to wetting by liquids with a surface tension lower than that of water. Interestingly, this parameter depends both on the surface structure and varies for the textures studied, and on the type of modifier. From here it becomes obvious that the self-hydrophobization layer differs from treatment with modifiers in terms of their ability to repel liquids of reduced polarity (Table 5).

All microtextured samples treated with stearic acid or OCTEO showed a comparable performance within errors. The surface of the sample “L”, which contained only a LIPSS pattern, turned out to be 2–3 times less resistant to the wetting transition for liquids with a low surface tension.

### 3.4. UV-Stability of Liquid-Repellent Properties

**Figure 7:**
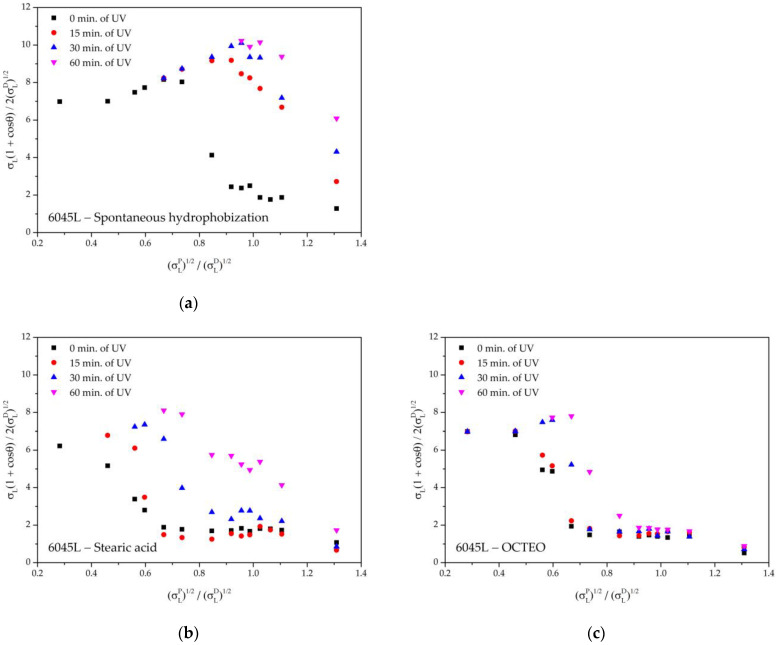
Owens–Wendt plots for the sample “6045L” under the influence of UV irradiation: (**a**) spontaneous hydrophobization; (**b**) treatment with stearic acid; (**c**) treatment with OCTEO.

To compare the UV-stability of the liquid-repellency of surfaces treated with stearic acid and OCTEO with those obtained after spontaneous hydrophobization, the sample “6045L” with a combination of micro- and nanotexture was selected. UV irradiation led to a loss of liquid-repellent properties, as can be seen from Figure 7 by the increased linearization of the Owens–Wendt plots with increasing exposure time. The reason for this was most likely photooxidation of thin organic layers of the modifier, which, in addition to laser ablation, also caused an increase in the proportion of polar fragments of molecules remaining on the surface. A similar effect has been reported on titanium surfaces, where a controlled wetting transition occurred after laser processing and exposure to a siloxane-contaminated atmosphere in a mild oven cycle. The sample was then returned to its initial hydrophilic state after UV irradiation [73] to illustrate the reversibility of the wetting transition. It is worth mentioning the general trends in the wetting transition on a variety of metal oxide surfaces (ZnO, TiO_2_, Bi_2_O_3_), described in detail in [74]. The hydrophobicity of the samples decreased after UV treatment, which was associated with the oxidation of the thin absorbed organic layer, but was restored in air or after heat treatment.

It is obvious that the samples after self-hydrophobization were less resistant to UV irradiation than the sample treated with stearic acid. The sample treated with OCTEO showed the best resistance. This can be explained by the fundamentally higher bond energy of the Si-O-Si and Si-C in organosilicon compounds than the C-C bond in hydrocarbons [75,76]. In addition, silicone polymers are known to have increased resistance to UV radiation compared to polyolefins [77]. In our experiments, the use of OCTEO provided a superhydrophobic state even after 60 min of UV irradiation, although an increase in polarity was still observed.

**Figure 8:**
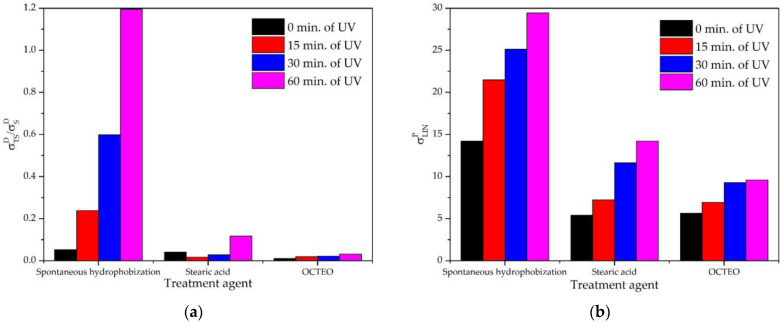
Derivative parameters of the Owens–Wendt plot for textured surfaces of the sample “6045L” under UV irradiation: (**a**) for the parameter $\sigma_{TS}^{D}$/$\sigma_{S}^{D}$; (**b**) for the parameter $\sigma_{LIN}^{P}$.

These changes can be most clearly illustrated by analyzing the derivative parameters $\sigma_{TS}^{D}$/$\sigma_{S}^{D}$ and $\sigma_{LIN}^{P}$ (Figure 8). UV exposure in the first 15 min already led to an increase in the parameter $\sigma_{TS}^{D}$/$\sigma_{S}^{D}$ up to 5 times in the case of self-hydrophobization, and after 60 min by 25 times (Figure 8a). Stearic acid turned out to be more stable as a modifier. After 15 min from the beginning of the photodegradation process, a slight decrease in this parameter was observed. According to the data of [78], this can be explained by the destruction of external excess layers of the modifier, weakly oriented towards the surface. After 60 min, this parameter increases only four times from the initial value. This parameter for OCTEO showed the best stability with virtually no changes.

However, the increase in surface layer polarity using OCTEO became evident when the change in $\sigma_{LIN}^{P}$ was taken into account (Figure 8b). After 60 min of exposure, it increased from 5 to 10 mN/m. Under the same conditions, for a sample treated with stearic acid, this value reached 13 mN/m, comparable to the initial value after self-hydrophobization. In the case of spontaneous hydrophobization, after an hour of exposure, a value of 28 mN/m was reached.

Thus, for all the surfaces studied, under the influence of UV irradiation, their polarity increased with a deterioration in liquid-repellent properties, including the water-repellency. Moreover, the stability of the layer obtained by spontaneous hydrophobization was significantly lower than that obtained by modification even with such a simple chemical substance as stearic acid. This result was also confirmed by the conclusions [79] regarding the instability of such a layer with environmental factors and, thus, casts doubt on the future practical use of such surfaces under conditions of prolonged exposure to sunlight. For outdoor applications, siloxane and possibly fluorinated modifiers are better suited, providing greater durability due to stronger interatomic bonds than hydrocarbon modifiers, such as oleic and stearic acids.

The appearance of an adsorbed layer after self-hydrophobization can also become a problem if subsequent chemical modification is intended after laser texturing. This layer can shield the access of the modifier to the treated surface, which requires its removal either by heating, as was conducted in this work, or by more energy-efficient UV treatment. On the other hand, without chemical modification, the adsorbed layer is spontaneously renewed upon contact with the atmosphere after any cleaning, restoring water-repellent properties up to superhydrophobicity. Similar processes occur among insects and plants, when the waxy layer on their surface can also be renewed after damage.

## 4. Conclusions

It has been shown that the use of the parameters $\sigma_{TS}^{D}$/$\sigma_{S}^{D}$ and $\sigma_{LIN}^{P}$ derived from the Owens–Wendt method allows an accurate comparison of the degree of liquid-repellency of textured surfaces, both those formed spontaneously after prolonged exposure to air, and those obtained by chemical treatment. It also makes it possible to track changes in the polarity of surfaces as they age under UV irradiation.

The liquid-repellency of femtosecond-laser-textured AISI 304 stainless steel surfaces after spontaneous hydrophobization has been shown to be significantly less resistant to UV irradiation than that of surfaces treated with modifiers, such as stearic acid and octyltrimethoxysilane.

The crucial role of surface topography in the formation of liquid-repellent properties was also demonstrated. The most effective turned out to be a texture containing microgrooves 45 µm-wide with a pitch of 60 µm on the surface of the “6045” sample, although not subjected to LIPSS processing, but replete with artifacts up to ~2 µm on the protrusions. The LIPSS nanopattern itself exhibited the least pronounced liquid-repellent properties, which were significantly enhanced by the formation of microgrooves.

## Figures and Tables

**Table 1:** Sample texture parameters.

Sample	Microgrooves		Nanopattern
“Flat St” (reference)	–		–
“L”	–	–	LIPSS
“6030L”	pitch 60 µm	width 30 µm	LIPSS
“10030L”	pitch 100 µm	width 30 µm	LIPSS
“6045L”	pitch 60 µm	width 45 µm	LIPSS
“6045”	pitch 60 µm	width 45 µm	–

**Table 2:** EDS analysis of element content in texture samples (wt. %).

Sample	Area	C	O	Si	Cr	Fe	Ni
6030L	1	3.47	13.8	0.27	14.65	59.29	8.52
	2	3.7	4.56	0.32	17.83	64.73	8.86
10030L	1	4.1	15.39	0.33	14.65	57.56	7.96
	2	4.31	4.61	0.38	18.05	64.69	7.97
6045L	1	3.19	15.24	0.59	14.86	58.15	7.98
	2	3.27	3.44	0.18	19.33	66.25	7.53
6045	1	9.2	21.4	0.7	13.65	47.58	7.47
	2	4.17	3.74	0.28	18.6	67.42	5.79

**Table 3:** Free surface energy and its component for the reference plate.

Surface Energy Component	Spontaneous Hydrophobization	Stearic Acid	OCTEO
$\sigma^{D}$, mN/m	31	25.5	25
$\sigma^{P}$, mN/m	7.2	2.6	1.2
σ, mN/m	38.2	28.1	26.2

**Table 4:** The $\sigma_{TS}^{D}$/$\sigma_{S}^{D}$ parameters of textured surfaces.

Surface Treatment	Sample L	Sample 6030	Sample 10030L	Sample 6045L	Sample 6045
Spontaneous hydrophobization	0.771	0.402	0.221	0.053	0.012
Stearic acid	0.934	0.088	0.088	0.042	0.031
OCTEO	0.132	0.052	0.066	0.011	0.015

**Table 5:** The $\sigma_{LIN}^{P}$ parameters of textured surfaces.

Surface Treatment	Sample L	Sample 6030	Sample 10030L	Sample 6045L	Sample 6045
Spontaneous hydrophobization	33.5	10.6	12.4	14.2	13.8
Stearic acid	16.2	5.2	4.1	5.4	4.9
OCTEO	10.3	4.5	4.3	5.7	7.22

## Data Availability

Data are contained within the article.
